# Investigation of RFLP Haplotypes β-Globin Gene Cluster in Beta-Thalassemia Patients in Central Iran

**Published:** 2019-04-01

**Authors:** Zahra Sajadpour, Zeinab Amini-Farsani, Majid Motovali-Bashi, Mitra Yadollahi, Farrokh Yadollahi

**Affiliations:** 1 Division of Genetics, Department of Biology, Faculty of Sciences, University of Isfahan, Isfahan, Iran; 2 Young Researchers and Elites Club, Shahrekord Branch, Islamic Azad University, Shahrekord, Iran; 3 Department of Operative Dentistry, School of Dentistry, Shahrekord University of Medical Sciences, Shahrekord, Iran; 4 Department of Anesthesiology, Clinical Research Development Unit, Kashani Hospital, Shahrekord University of Medical Sciences, Shahrekord, Iran

**Keywords:** Haplotype, Beta-thalassemia, c.315+1G>A, c.92+5G>C, c.93-21G>A

## Abstract

**Introduction**: Beta-thalassemia is one of the most prevalent inherited blood diseases among Iranians. The aim of this study was to elucidate the chromosomal background of beta-thalassemia mutations in Esfahan province, Iran.

**Materials and Methods:** In this study, we investigated three frequent mutations (c.315+1G>A, c.93-21G>A and c.92+5G>C in β-globin gene, the frequency of RFLP haplotypes, and LD between markers at *β*-globin gene cluster) in 150 beta-thalassemia patients and 50 healthy individuals. The molecular and population genetic investigations were performed on RFLP markers *Hin*dIII in the c.315+1G>A of *Gγ* (*Hin*dIIIG) and *Aγ* (*Hin*dIIIA) genes, *Ava*II in the c.315+1G>A of β-globin gene and *Bam*HI 3' to the β-globin gene. All statistical analyses were performed using Power Marker software and SISA server.

**Results:** Fifty percent of beta-thalasemia patients were associated with these mutations. Haplotype I was the most prevalent haplotype among beta-thalassemia patients (39.33%) and normal individuals (46%). The commonest c.315+1G>A mutation in our population was tightly linked with haplotype III (43.75%) and haplotype I (31.25%). The second prevalent mutation, c.92+5G>C, was 90%, 6.66%, and 3.33% in linkage disequilibrium with haplotypes I, VII, and III, respectively. The c.93-21G>A mutation indicated a strong association with haplotype I (80%).

**Conclusion:** Our study participants like beta-thalassemia patients from Kermanshah province was found to possess a similar haplotype background for common mutations. The emergence of most prevalent mutations on chromosomes with different haplotypes can be explained by gene conversion and recombination. High linkage of a mutation with specific haplotype is consistent with the hypothesis that chromosomes carrying beta-thalassemia mutations experienced positive selection pressure, probably because of the protection against malaria experienced by beta-thalassemia carriers.

## Introduction

 Thalassemia patients comprise approximately 5% of global population. The gene frequency of beta thalassemia is high in Iran and varies in different regions of the country^1^. In Isfahan, located around Zayanderud, its frequency has been estimated up to approximately 8%^2^. Clinical complications and mental difficulties which are prevalent in thalassemia patients, the costs expended to treat these patients, and high frequency of beta- thalassemia gene in Isfahan highlight the necessity of further research^2^^,^^3^. The use of genetic markers may be beneficial when mutation is undetected^4^. Sequencing and direct detection of mutations is a time-consuming process due to the large number of known mutations and potential new mutations in the β-globin gene. Therefore, alternative methods of genetic diagnosis are according to linkage studies by means of polymorphic markers linked to the β-globin gene region^5^. Although markers could be independently investigated, analysis of markers as haplotypes is empirically more informative. The haplotypes with 5% or higher frequency are considered as population-identifying^6^. In some subpopulations, a limited number of RFLP-haplotypes of β-globin gene cluster has indicated non-random association (high linkage) with specific beta -thalassemia mutations^7^^,^^8^. Analysis of beta haplotype is useful for identification of new mutations in β-globin gene, prenatal diagnosis, and epidemiological studies. Also, it should be extensively done to detect the mechanism and origin of beta-thalassemia mutations and to implement novel diagnostic and therapeutic strategies^9^. Prenatal diagnosis of β-globin gene disorders using molecular markers depends on linkage disequilibrium in the β-globin gene mutations and the adjacent markers. Failure to consider this could lead to error in diagnosis. Recombination hot spots reduce linkage disequilibrium and breakdown in the pattern of haplotype blocks in the short distances as much as 1-2 kb. The β-globin gene cluster contains one of the first known hot spots in humans^10^. Therefore, it is necessary to measure the LD between the used markers alongside the estimation of haplotype frequency in a population.

Iran’s population consists of various subpopulations, and therefore each region could have an independent set of mutations and haplotypes. To the best of our knowledge, no study has been yet conducted to investigate the association of the haplotypes with beta thalassemia mutations in Isfahan. The present study was conducted to determine the frequency of *Hin*dIIIA- *Hin*dIIIG- *Ava*II- *Bam*HI haplotypes and their association with the three prevalent beta- thalassemia mutations among the population of Isfahan Province.

## MATERIALS AND METHODS

**Study population**

The patient group consisted of 150 unrelated beta-thalassemia major patients who referred to the Thalassemia Ward of Seyed al-Shohada Hospital of Isfahan. The control group included 50 healthy individuals from the same area. The necessary data, including demographic characteristics, family history and hemoglobin level (from electrophoresis results in patients’ medical file) were recorded in a questionnaire, and blood samples were taken with the patients’ consent. The exclusion criteria included patients with other anemia due to other causes.

**Β- globin genotyping and haplotyping**

Blood samples were collected in sterile tubes containing EDTA, and DNA was extracted from whole blood using salting out method^11^. Quality of extracted DNA was evaluated according to 260/280 absorbance ratio, measured by Nano Drop spectrometer (Thermo Scientific, Waltham, MA, USA). Afterward, prevalent mutations, c.315+1G>A; c.92+5G>C; c.93-21G>A, were investigated by ARMS-PCR using primers in Old study^12^. For analysis of haplotype markers [*Hin*dIII in the c.315+1G>A of *Gγ* (*Hin*dIIIG) and *Aγ* (*Hin*dIIIA) genes, *Ava*II in c.315+1G>A of the he β-globin gene and *Bam*HI 3' to the β-globin gene], a part of genomic DNA containing the fragment of interest was amplified by PCR and digested with the appropriate restriction enzyme. The primers used in this study had already been used in a previous study^13^. 

**Statistical analysis**

All statistical analyses were performed by Power Marker software and SISA server.

## Results

**Β-globin genotype**

In the present study, three prevalent mutations c.315+1G>A, c.93-21G>A and c.92+5G>C were screened in 150 beta-thalassemia patients. Sixty of 150 patients were associated with mentioned mutations. The frequencies of mutations are shown in Table1.

**Table 1:** Frequency of mutations

Mutation	Number patients (%)
c.315+1G>A / c.315+1G>A	40 (26.66%)
c.92+5G>C / c.92+5G>C	15 (10%)
c.93-21G>A / c.93-21G>A	5 (3.33%)
Total	60

**Β-globin haplotype**

**Figure 1:**
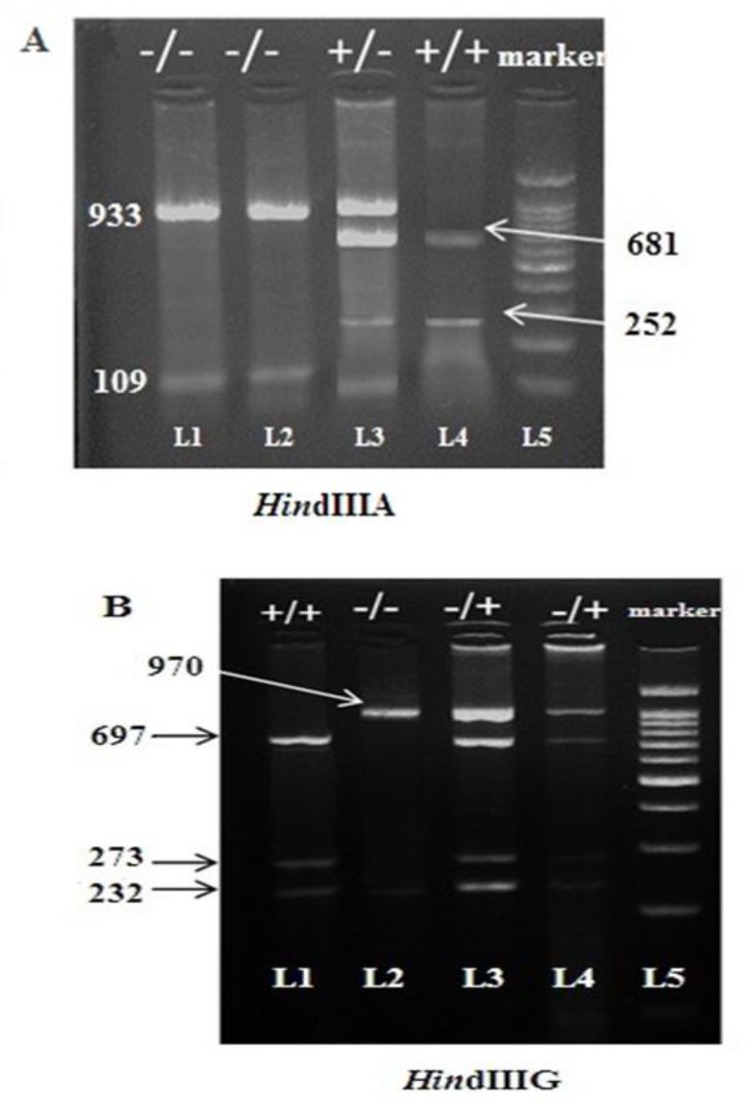
***Hin*** **dIII PCR–RFLP products on agarose gel.** **A.** ***Hin*dIIIA.** L1 and L2 showing the undigested samples, L3 showing the heterozygosity and L4 showing the homozygousity. L5 is 1000 bp DNA ladder. For homozygous individuals (-/-) two undigested band (933 bp and 109 bp) were visible. Four digested bands (933 bp, 681 bp, 252 bp and 109 bp) were seen in heterozygous individuals, whereas for homozygous patients (+/+) three digested bands (681 bp, 252 bp and 109 bp) were detected. **B.** ***Hin*dIIIG.** L1 showing the homozygousity, L2 showing the undigested sample, and L3 and L4 showing the heterozygosity. L 5 is 1000 bp DNA ladder. For homozygous individuals (-/-) two undigested band (970 bp and 232 bp) were visible. Four digested bands (970 bp, 697 bp, 273 bp and 232 bp) were seen in heterozygous individuals, whereas for homozygous patients (+/+) three digested bands (697 bp, 273 bp and 232 bp) were detected.

**Figure 2:**
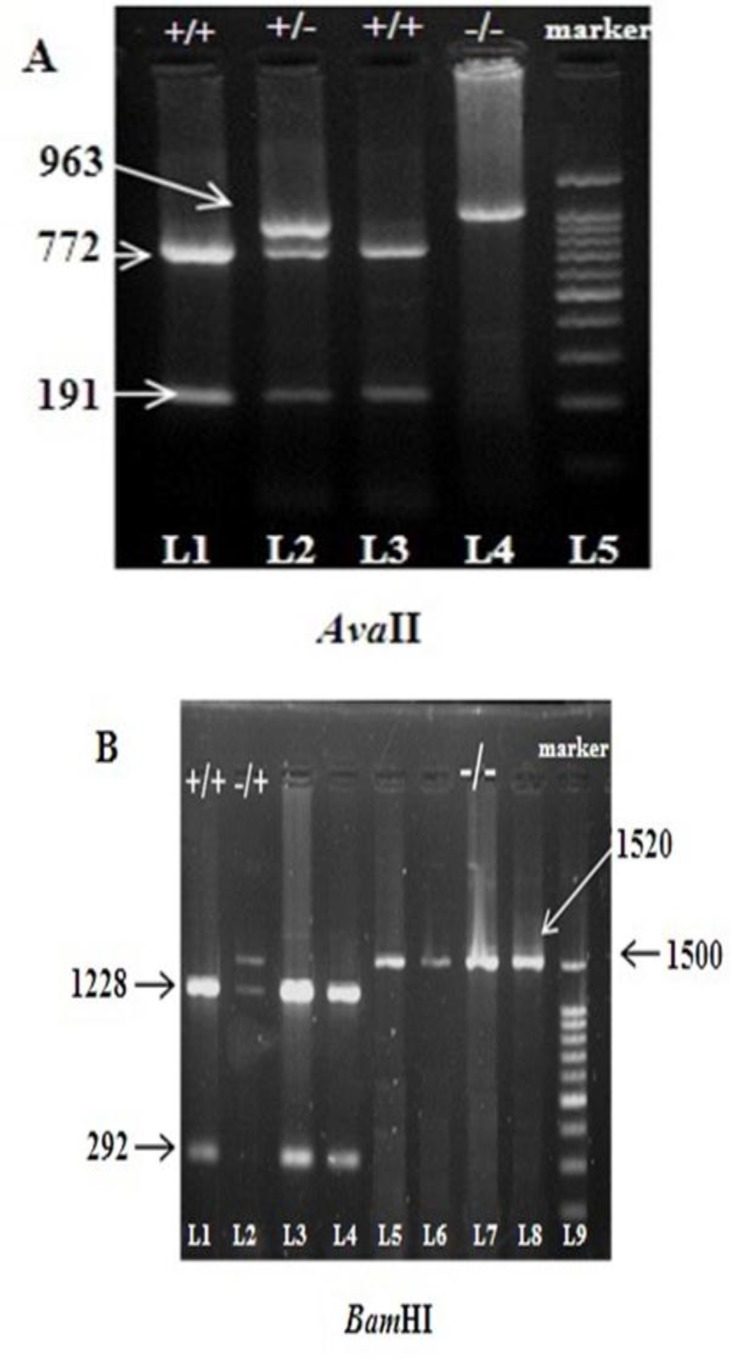
*Ava*II and *Bam*HI PCR–RFLP products on agarose gel. **A.** ***Ava*II.** L1 and L3 showing homozygousity, L2 showing the heterozygosity and L4 showing the undigested sample. L5 is 1000 bp DNA ladder. For homozygous individuals one undigested band (963 bp) was visible. Three digested bands (963 bp, 772 bp, and 191 bp) were seen in heterozygous individuals, whereas for homozygous patients (+/+) two digested bands (772 bp, and 191 bp) were detected. **B.** ***Bam*HI.** L1 showing the homozygousity, L2 showing the heterozygosity. L7 showing undigested sample. L5 is 1000 bp DNA ladder. For homozygous individuals (-/-), one undigested band (1520 bp) was visible. Three digested bands (1520 bp, 1228 bp, and 292 bp) were seen in heterozygous individuals, whereas for homozygous patients (+/+) two digested bands (1228 bp and 292 bp) were detected.

Haplotype analysis was performed in 150 beta- thalassemia carriers and 50 healthy individuals using Power Marker software, and 6 different haplotypes were identified. Orkin et al’s naming of the haplotypes was used in the present study. The results of the β-globin gene cluster haplotype analysis are shown in Figures 1 and 2. 

As shown in Table 2, haplotype I was the most prevalent haplotype followed by haplotypes III, VII and II among healthy and beta-thalassemia chromosomes with the frequency of 46% vs. 39.33%, 26% vs. 32.66%, 16.66% vs. 18.33% and 3% vs. 8.33%, respectively. Overall, haplotypes I and III comprised 70% of the haplotypes. Haplotypes I, III and VII as well as haplotype II (8.3%) with over 5% frequency in beta-thalassemia chromosomes could be considered as identifying haplotypes in the population (Table 2). The most prevalent mutation, c.315+1G>A, in Isfahan province exhibited 43.75% in linkage with haplotype III and 31.23% with haplotype I, while haplotypes III and I were 26% and 46% linkage with healthy chromosomes, respectively. Interestingly, the most prevalent mutation (c.315+1G>A) in both most prevalent haplotypes (I and III) was the most frequent. c.315+1G>A was also linked with haplotypes IV and VII but less than the first two haplotypes. c.92+5G>C mutation was in linkage with haplotype I (90%), haplotype VII (6.66%) and haplotype III (3.33%). Also c.93-21G>A exhibited 80% in linkage with haplotype I and 20% with haplotype III (Table 3).

**Table 2:** Comparison of frequency of haplotypes between healthy and Beta-thalasemia chromosomes

Name	Haplotype	β^A^ Frequency	β^thal^ Frequency
**I**	**+ + - -**	46.0%	** 39.33%**
**III**	**- + + -**	26.0%	**32.66%**
**VII**	**+ - - -**	16.66%	**18.33%**
**II**	**+ + + +**	8.33%	**3%**
**VI**	**+ - + +**	1.6%	**5%**
**IV**	**- - + -**	0	**1.6%**
**Total** **(chromosome)**		100	**300**

**Table 3:** Haplotype frequency of three prevalent mutations

Name	Haplotype	c.315+1G>A	c.92+5G>C	c.93-21G>A
**III**	**- **+ +** -**	43.75	3.33%	**20%**
**I**	+ + **- -**	31.25	90%	**80%**
**VII**	+ **- - -**	20	6.66%	**0**
**IV**	**-** **- **+ **-**	5	0	**0**
**Total** **Chromosome**		80	30	**10**

**Linkage disequilibrium**

After polymorphic markers were genotyped in the studied population, linkage disequilibrium was assessed by Power Marker software. The presence of mutant alleles in the studied population was thought to falsely cause an increase in the calculated LD between the markers. To remove this problem, D' and *P*-value were calculated for chromosomes of 50 healthy individuals. As shown in Table 4, D' was 0.8869 (*P*=0) between *Hin*dIIIG and *Hin*dIIIA and 0.64 (*P*=0) between *Bam*HI and* Ava*II (Table 4).

**Table 4:** Calculation of D' and Fisher's exact test P value by Power Marker software

Marker 1	Marker 2	D'	*p*-value
**HindIIIA**	*Hin*dIIIG	0.8869	**0.0**
**HindIIIA**	*Ava*II	0.2704	**0.267**
**HindIIIG**	*Ava*II	0.106	**0.2**
**HindIIIA**	*Bam*HI	0.0283	**0.68**
**HindIIIG**	*Bam*HI	0.0032	**0.9**
**BamHI**	*Ava*II	0.64	**0.0**

## Discussion

 Beta-thalassemia is one of the most common inherited blood diseases in Iran. The objective of this study was to evaluate the β-globin gene cluster haplotypes of the beta-thalassemia mutations in Esfahan province. In the present study, the three common beta-thalassemia mutations among 150 Iranian patients with beta-thalassemia have been characterized. The most common mutation was c.315+1G>A (26.66%), followed by c.92+5G>C (10%) and c.93-21G>A (3.33%). The c.315+1G>A mutation was found to be the most frequent mutation in the Kurdistan, Azarbayjan-Sharghi, and Fars provinces of Iran as well as most of the Arab countries ^14^^-^^16^.

In haplotype analysis, we identified 6 different haplotypes that haplotype I was the most prevalent haplotype (46%), followed by haplotypes III (26%), VII (16.66%), II (8.33%), and VII (1.6%) in chromosomes of beta-thalassemia patients. Consistent with our results, Rahimi's report in the western of Iran confirmed that haplotype I was the most frequent haplotype among beta thalassemia major individuals and normal controls (35.7% and 42.9%, respectively), followed by haplotype III (28.6%) in beta-thalassemia patients^12^. Similarly, haplotype I was the most frequent haplotype among beta-thalassemia patients from Mediterranean area^17^. Haplotypes I, II and III were also the most common in Italy, Greece, India and Turkey^18^. In Fars Province, haplotype I was derived as the most frequent haplotype, followed by haplotypes V and III in beta-thalassemia carriers and healthy individuals with a frequency of 46.2% vs. 43%, 15.4% vs. 3.8% and 7.7% vs. 15.4%, respectively^19^. Since Haplotype I is the most frequent haplotype among beta-thalasemia patients and healthy individuals, it implies that beta- thalassemia mutations could have arisen from common chromosomal backgrounds in the population. However, this could be partially explained by selection pressure or gene flow^1^. 

The strong linkage disequilibrium of beta gene cluster haplotypes with prevalent mutations has been reported previously^20^^-^^22^. In our population, c.315+1G>A mutation is in linkage disequilibrium with haplotypes III (43.75%), I (31.25%), VII (20%), and IV (5%), respectively*. *It can be suggested that the high frequency of the c.315+1G>A mutation resulted in the high prevalence of haplotype III in our study. Rahimi et al.’s report in Kermanshah province has indicated that c.315+1G>A mutation was tightly linked with haplotype III which is in line with our results, representing the same origin of this mutation in two regions^12^.

Conversely, this mutation was linked to haplotypes I and III in Hormozgan and Fars provinces^19^. Mansoori et al. have shown that this mutation was predominantly associated with haplotype IV in Azarbayjan-Sharghi province^16^. The association of this mutation with haplotypes III and V among the Turkish population ^23^ and with haplotypes I and III among the Palestinian ^24^ and Lebanese ^25^ populations have been reported, respectively.

The mutation c.92+5G>C was firstly reported from China and India and is the most prevalent mutation in the UAE. However, this mutation in the Arab population is found on a haplotype of β-globin which is different from that in India and hence is thought to have a different origin^15^. In Brazil, c.92+5G>C is linked with haplotypes I and V^26^. Most alleles of Indian with this mutation have haplotype VII^27^. In Kermanshah population, this mutation was tightly linked with c.315+1G>A (100%) ^12^. In the present study, c.92+5G>C mutation indicates a strong association with haplotypes I (90%) and a weak association with haplotypes VII (6.66%) and III (3.33%), respectively. Moreover, c.93-21G>A mutation was 80% and 20% in linkage disequilibrium with haplotypes, I and III, respectively. In Kermanshah province, c.93-21G>A mutation was found to be linked to haplotypes I, IX, A and atypical^12^. Previous studies show that this mutation is associated with haplotype I in Lebanon, Jewish, Turkey and Algeria populations ^28^^-^^30^, and with haplotypes XIII and I in Mediterranean region^17^.

Calculation of LD among the markers demonstrated that between the markers of 5', i.e., HindIIIA and HindIIIG, there was linkage disequilibrium, but these markers did not have linkage disequilibrium with any markers of 3'. Besides, there was a high linkage between markers of 3', i.e., BamHI and AvaII (D'=0.64, p=0). These findings indicate that recombination hotspot is located somewhere between these two classes of markers, causing separation of linkage between them. In this report, the presence of hotspot and lack of LD among sub- haplotypes 5' and 3' were confirmed. These findings are consistent with Rahimi et al. study in Kermanshah, western of Iran^12^, Yavarian et al. study in Hormozgan, south of Iran ^31^, Rahimi et al. study in Fars, southwestern of Iran^32^. Moreover, the presence of hotspot and lack of LD among sub- haplotypes 5' and 3' have been confirmed in many studies worldwide. The rate and pattern of LD vary in different populations, which relate to the factors associated with genetic drift, migration and so on.

The emergence of most common mutations on chromosomes with different haplotypes can be explained by gene conversion and recombination^12^. However, all mutations are created on the same healthy chromosomal area. It follows that the most prevalent haplotypes in patients and healthy individuals are the same. This argument implies that the chromosomes with beta-thalassemia mutations have a positive selection pressure which is tolerated by carriers probably because of protection against malaria. The wide variety of haplotypes linked with a common mutation most probably represents that the mutation of interest is one of the oldest mutations of the area with adequate time for recombination or the mutation has been created independently on several haplotype areas. The linkage of the haplotype with the mutation is virtually similar in different areas, contradicting the mutations' independent development on several haplotype areas. The creation of multiple haplotype linkages with the mutation may simply be due to gene conversion events in globin genes. The present study is consistent with Rahimi et al. study in Kermanshah^12^, potentially indicating the same origin of these mutations in the two regions. 

## CONCLUSION

 In this study, we first reported β-globin gene cluster haplotypes association with a common mutation in Esfahan province in Iran. Our study participants like beta-thalassemia patients from Kermanshah province was found to possess a similar haplotype background for common mutations. Identification of the most prevalent mutations on different haplotype backgrounds can be explained by a variety of gene conversion and recombination events. High linkage of a mutation with specific haplotype is consistent with the hypothesis that chromosomes carrying beta- thalassemia mutations experienced positive selection pressure, probably because of the protection against malaria experienced by beta- thalassemia carriers.

## References

[1] Rahimi Z, Vaisi Raygani A, Merat A (2006). Thalassemic mutations in Southern Iran. Iran J Med Sci.

[2] Derakhshandeh-Peykar P, Hourfar H, Heidari M (2008). The Spectrum of β -thalassemia Mutations in Isfahan Province of Iran. Iran J Public Health.

[3] Senol SP, Tiftik EN, Unal S (2016). Quality of life, clinical effectiveness, and satisfaction in patients with beta thalassemia major and sickle cell anemia receiving deferasirox chelation therapy. J Basic Clin Pharm.

[4] Raza SH, Waseem Shoaib M, Mubeen H (2016). Genetic Markers: Importance, uses and applications. IJSRP.

[5] Gupta A, Sarwai S, Pathak N (2008). Beta-globin gene mutations in India and their linkage to β-haplotypes. Int J Hum Genet.

[6] Gattepaille LM, Jakobsson M (2012). Combining markers into haplotypes can improve population structure inference. Genetics.

[7] Falchi A, Giovannoni L, Vacca L (2005). Beta-globin gene cluster haplotypes associated with beta-thalassemia on Corsica island. Am J Hematol.

[8] Atweh GF, Forget BG (1986). Identification of a beta-thalassemia mutation associated with a novel haplotype of RFLPs. Am J Hum Genet.

[9] Öztürk O, Atalay A, Köseler A (2007). Beta globin gene cluster haplotypes of abnormal hemoglobins observed in Turkey. Turk J Haematol.

[10] Wood ET, Stover DA, Slatkin M (2005). The β-globin recombinational hotspot reduces the effects of strong selection around HbC, a recently arisen mutation providing resistance to malaria. Am J Hum Genet.

[11] Miller SA, Dykes DD, Polesky HF (1988). A simple salting out procedure for extracting DNA from human nucleated cells. Nucleic Acids Res.

[12] Rahimi Z, Muniz A, Akramipour R (2009). Haplotype analysis of beta thalassemia patients in Western Iran. Blood Cells Mol Dis.

[13] Lee YJ, Park SS, Kim JY (2002). RFLP Haplotypes of-Globin Gene Complex of-Thalassemic Chromosomes in Koreans. J Korean Med Sci.

[14] Najmabadi H, Karimi-Nejad R, Sahebjam S (2001). The beta-thalassemia mutation spectrum in the Iranian population. Hemoglobin.

[15] Zahed L (2001). The Spectrum of beta-thalassemia Mutations in the Arab Populations. J Biomed Biotechnol.

[16] Kotea N, Ramasawmy R, Lu CY (2000). Spectrum of β-Globin Gene Mutations and β-Thalassemia Haplotype Analysis among the Iranian Azeri Turkish Population. Am J Hematol.

[17] Orkin SH, Kazazian HH Jr, Antonarakis SE (1982). Linkage of beta-thalassemia mutations and beta-globin gene. Nature.

[18] Antonarakis SE, Boehm CD, Giardina PJ (1982). Nonrandom association of polymorphic restriction sites in the β-globin gene cluster. Proc Natl Acad Sci U S A.

[19] Rahimi Z, Merat A, Akhzari M (2005). β-Globin Gene Cluster Haplotypes in Iranian Patients with β-Thalassemia. IJHOSCR.

[20] Hashemi-Soteh MB, Mousavi SS, Tafazoli A (2017). Haplotypes inside the beta-globin gene: use as new biomarkers for beta-thalassemia prenatal diagnosis in north of Iran. J Biomed Sci.

[21] Rozitah R, Nizam MZ, Nur Shafawati AR (2008). Detection of beta-globin gene mutations among Kelantan Malay thalassaemia patients by polymerase chain reaction restriction fragment length polymorphism. Singapore Med J.

[22] Hockham C, Piel FB, Gupta S (2015). Understanding the contrasting spatial haplotype patterns of malaria-protective β-globin polymorphisms. Infect Genet Evol.

[23] Diaz-Chico JC, Yang KG, Stoming TA (1988). Mild and severe beta-thalassemia among homozygotes from Turkey: identification of the types by hybridization of amplified DNA with synthetic probes. Blood.

[24] El-Latif MA, Filon D, Rund D (2002). The beta +-IVS-I-6 (T-->C) mutation accounts for half of the thalassemia chromosomes in the Palestinian populations of the mountain regions. Hemoglobin.

[25] Makhoul NJ, Wells RS, Kaspar H (2005). Genetic heterogeneity of Beta thalassemia in Lebanon reflects historic and recent population migration. Ann Hum Genet.

[26] Martins JT, Bordin S, de Albuquerque DM (2005). Dnase I hypersensitive site 3' to the beta globin gene cluster containing two TAA insertions and a G>A polymorphism is predominantly associated with the beta+ thalassemia IVS-I-6 (T>C) mutation. Hemoglobin.

[27] Kukreti R, Dash D, E VK (2002). Spectrum of-Thalassemia Mutations and Their Association With Allelic Sequence polymorphisms at the beta-globin gene cluster in an Eastern Indian population. Am J Hematol.

[28] Zahed L, Qatanani M, Nabulsi M (2000). β-thalassemia mutations and haplotype analysis in Lebanon. Hemoglobin.

[29] Zahed L, Demont J, Bouhass R (2002). Origin and history of the C.93-21G>A and codon 39 beta thalassemia mutations in the Lebanese population. Hum Biol.

[30] Bahadır A, Öztürk O, Atalay A (2009). Beta globin gene cluster haplotypes of the beta thalassemia mutations observed in the Denizli province of Turkey. Turk J Haematol.

[31] Yavarian M, Karimi M, Bakker E (2004). Response to hydroxyurea treatment in Iranian transfusion-dependent b-thalassemia patients. Haematologica.

[32] Rahimi Z, Karimi M, Haghshenas M (2003). Beta globin gene cluster haplotype in cycle cell patient from Southwest Iran. Am J Hematol.

